# mHealth Self-Monitoring Model for Medicine Adherence of Patients With Diabetes in Resource-Limited Countries: Structural Equation Modeling Approach

**DOI:** 10.2196/49407

**Published:** 2023-10-23

**Authors:** Mmamolefe Kgasi, Bester Chimbo, Lovemore Motsi

**Affiliations:** 1 Faculty of ICT Tshwane University of Technology Pretoria South Africa; 2 School of Computing University of South Africa Johannesburg South Africa

**Keywords:** diabetes, mobile health, mHealth, self-monitoring, self-management, chronic diseases, health care provision

## Abstract

**Background:**

The COVID-19 pandemic has led to serious challenges and emphasized the importance of using technology for health care operational transformation. Consequently, the need for technological innovations has increased, thus empowering patients with chronic conditions to tighten their adherence to medical prescriptions.

**Objective:**

This study aimed to develop a model for a mobile health (mHealth) self-monitoring system for patients with diabetes in rural communities within resource-limited countries. The developed model could be based on the implementation of a system for the self-monitoring of patients with diabetes to increase medical adherence.

**Methods:**

This study followed a quantitative approach, in which data were collected from health care providers using a questionnaire with close-ended questions. Data were collected from district hospitals in 3 South African provinces that were selected based on the prevalence rates of diabetes and the number of patients with diabetes treated. The collected data were analyzed using smart partial least squares to validate the model and test the suggested hypotheses.

**Results:**

Using variance-based structural equation modeling that leverages smart partial least squares, the analysis indicated that environmental factors significantly influence all the independent constructs that inform patients’ change of behavior toward the use of mHealth for self-monitoring of medication adherence. Technology characteristics such as effort expectancy, self-efficacy, and performance expectancy were equally significant; hence, their hypotheses were accepted. In contrast, the contributions of culture and social aspects were found to be insignificant, and their hypotheses were rejected. In addition, an analysis was conducted to determine the interaction effects of the moderating variables on the independent constructs. The results indicated that with the exception of cultural and social influences, there were significant interacting effects on other independent constructs influencing mHealth use for self-monitoring.

**Conclusions:**

On the basis of the findings of this study, we conclude that behavioral changes are essential for the self-monitoring of chronic diseases. Therefore, it is important to enhance those effects that stimulate the behavior to change toward the use of mHealth for self-monitoring. Motivational aspects were also found to be highly significant as they triggered changes in behavior. The developed model can be used to extend the research on the self-monitoring of patients with chronic conditions. Moreover, the model will be used as a basic architecture for the implementation of fully fledged systems for self-monitoring of patients with diabetes.

## Introduction

### Background

Many resource-limited countries, especially those in Africa, face health inequalities that have led to a triple burden of traditional, infectious, and chronic diseases [1]. The health inequality gap manifests in many ways, including the lack of medical and food accessibility, poor sustainable income leading to a lack of meeting daily needs, poor alignment of innovation with disease burdens, and underserved communities that limit the monitoring of patients who are chronically ill [2]. The literature indicates that chronic diseases, both communicable and noncommunicable, have contributed to many deaths and have caused a great deal of strain on health care systems in resource-limited countries [3,4]. In addition, patients with chronic conditions often become traumatized while facing an incurable illness. This is detrimental to their medical compliance and ultimately contributes to their death [1,5].

Many resource-limited countries face unemployment, which has led to migration from rural to urban areas [1]. With the increase in urbanization, more than 56% of the people in resource-limited countries now live in towns and cities. However, socioeconomic disparities and other factors such as work stress, environmental factors, and low income generation negatively impact the lives of these new town or city immigrants [4,6]. Because of the high cost of living in urban areas, people’s lifestyles have changed drastically. This has contributed to the increasing prevalence of both communicable and noncommunicable diseases as well as infectious diseases [2]. Diseases such as cardiovascular disease, chronic obstructive pulmonary diabetes, tuberculosis, and HIV have become more prevalent in resource-limited countries [5].

The World Health Organization report of 2020 indicated that diabetes and other noncommunicable diseases resulted in over 16% of all deaths in South Africa in 2016 [7]. This high prevalence was primarily due to the lack of awareness of diabetes, lack of access to adequate health care, and inability to follow medical prescriptions. It is pertinent to mention that this is taking place at a time when South Africa is spending a great deal on health care, with diabetes being on its political agenda [5]. The COVID-19 pandemic has strained the health care systems in multiple countries. The high prevalence of chronic diseases in resource-limited countries has further exacerbated. Moreover, many resource-limited countries, including South Africa, had already faced numerous burdens of disease before the emergence of COVID-19. For example, for decades, malaria and measles have been traditional health hazards in equatorial regions [1,6]. These multiple burdens of disease demand technological interventions such as mobile health (mHealth) to empower patients to take control of their own health.

Therefore, it is important to provide patients who consume medicines regularly with technology-based self-monitoring. An mHealth self-monitoring application system will serve as a reminder to timely attend to patients’ health needs. Those living with a chronic disease often give up trying because of anxiety [1,8]. The World Health Organization [7] showed that inconsistency in drug intake is a major cause of high mortality rates in people with chronic diseases. This is because routine and timely medication administration suppresses symptoms and other maladies that complicate diabetic conditions. Low immunity levels make these ailments and symptoms more likely to worsen the patient’s condition owing to poor adherence to medicine [9,10]. The use of technology to elicit behavioral changes for self-monitoring of complicated ailments such as diabetes has been deemed rewarding by several researchers [11,12].

Regardless of the country’s economic status, health care responsiveness is of paramount importance. To ensure equal access to health care [13], countries should find better ways of allowing all citizens access to health care, regardless of their location. A key component of this is providing health services closer to communities, especially those outside the constant reach of health care workers. According to the study by Achoki et al [14], increasing the accessibility of health care systems is crucial to bridging the urban-rural divide by solving health care inequality. Because of efforts to improve health care system accessibility and availability, information technology has been used to provide health care [10,15,16].

### mHealth for Self-Monitoring of Patients

The use of technology has become ubiquitous and pervasive in facilitating the monitoring and provision of health care. According to the study by Islam et al [17], mHealth has gained traction as a powerful tool for monitoring both patients and medical personnel. These researchers have indicated that mHealth technology has been leveraged to manage chronic diseases such as diabetes, hypertension, and other cardiovascular ailments, as well as maternal health and psychological disorders. Researchers such as Abduo et al [18] and Lin et al [19] have also observed that mHealth has the potential to improve the efficiency and effectiveness of health care management for patients by health care workers and health system managers. This is accomplished by offering real-time guidelines, referral services, and procedures. When mHealth is used effectively, it can also serve as a reminder system for patients to self-manage and administer their medication, thereby improving medication adherence [9].

As a communication tool, mHealth has been used to distribute information via simple messages (SMS text messages) and alerts via other reminders, enabling patients to adhere to their treatment regimen. However, because medication adherence involves lifestyle changes, including behavioral adjustment, patients require positive incentives for such behavioral changes [20]. Hence, as self-management and administration of medical prescriptions are affected by behavior, an mHealth system that works as a reminder must include a component that considers human behavior [9]. It is imperative that technological applications such as mHealth be integrated with human behavior in the form of a persuasive model. This implies that mHealth alone may not be sufficient as a communication tool, and the behavioral aspects of patients must be considered. This also requires contextualization, thus considering patients’ cultural and social backgrounds [5,21].

### Theoretical Perspectives of mHealth Self-Monitoring

According to Fogg [22], during any human-computer interaction, humans may respond to computers as though they are human. This could be because humans naturally respond to social presence by expressing empathy, being angry, or participating in social activities. Humans are hardwired to respond to signals in the environment in which they live; such responses are instinctive rather than rational. Nass et al [23] argued that computers serve as persuasive social actors by rewarding individuals with positive feedback. This is accomplished by modeling the desired behavior or attitudes and thereafter providing psychological support. Furthermore, Fogg [24] observed that humans also experience social presence, whether by empathizing or feeling angry or by performing social actions. Fogg [24] added that computing products trigger individual automatic responses because of the social cues provided by them. The author emphasized that factors such as motivation, ability, and prompting are required as simultaneous actions for a given behavior to occur.

Researchers such as Larbi et al [11] and Reidy et al [25] noted that for self-monitoring, a patient must be motivated to perform the actual behavior of taking medicine. The motivation may arise from the patient’s yearning to live a stress-free life and have a social connection. This implies that such an individual must have the ability to easily comply with whatever the mHealth app is suggesting. This could be termed as self-efficacy and ease of use. This signifies the importance of including the acceptance and use of technology factors in the mHealth self-monitoring model. In addition, Fogg [24] emphasized that persuasive technology involves the incorporation of insights from psychology into the design of products. This applies to mobile apps and wearables that modify people’s habits and beliefs; factors such as motivation and ability should be considered in the design process.

### The Conceptual Model

To inform the acceptance and use of technology, various theories and models have been developed, most of which are extensions and modifications of the old popular models of the theory of reasoned action [26,27], diffusion of innovation [28], the technology acceptance model [29], and the Unified Theory of Acceptance and Use of Technology (UTAUT) [30]. According to these models, although heterogeneous factors may lead to the success of technological innovation, actual behavior, such as acceptance and use, is generally preceded by behavioral intention (BI) [31,32]. In general, newer theories and models have been developed because of the shortcomings of their predecessors.

Several commonly used theories and models for predicting technology acceptance and use are extensions of previous models, and their constructs are essentially the same. Similarly, the behavioral change wheel model [33], which has been widely used for behavioral change interventions, was developed by unifying 19 frameworks, 9 intervention functions, and 7 policy categories for classifying behavioral change. In contrast, the theoretical domains framework (TDF) [34] recommended for understanding behavioral change processes during the implementation of evidence-based interventions was also developed from a synthesis of psychological theories [35,36]. The TDF has 14 domains: social influences (SIs); environmental context and resources; social and professional role and identity; beliefs about capabilities; optimism; intentions; beliefs about consequences; reinforcement; emotion; knowledge; cognitive and interpersonal skills; memory, attention, and decision processes; behavioral regulation; and physical skills. These domains have been widely used to study how behavior changes when evidence-based care is implemented, which cuts across those of acceptance and use theories relating to behavioral change interventions.

**Figure 1:**
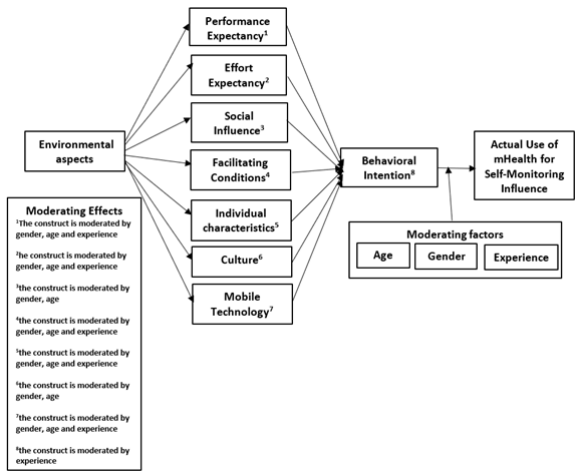
The conceptual model. mHealth: mobile health.

Both the behavioral change wheel and TDF, along with acceptance and use theories, have been offered as theoretical approaches in the design of tools for interventions aimed at changing behavior [37]. Therefore, a conceptual model for exploring implementation problems for enhancing health care practices should clarify a wide range of potential mediators of behavioral change. This study used an extended version of the UTAUT, underpinning the design of its conceptual model. The extended model of the UTAUT encompasses behavioral change intervention aspects, technology characteristics that promote self-efficacy and ease of use, as well as individual characteristics (IC) that support patients’ motivation. In addition, the conceptual model considers age, gender, experience, and motivation as moderating variables. The conceptual model is illustrated in Figure 1.

### Hypothesis Development and Coding

#### Environmental Aspects

Environmental aspects refer to the environment in which the patient lives. Among these factors are network availability, government policies and standards for the use of mHealth, affordability by patients of the mHealth system, availability of relevant infrastructure to support mHealth systems, as well as educational support for those unfamiliar with mHealth systems [38]. Environmental aspects have been proposed to influence the other independent variables of performance expectancy (PE), effort expectancy (EE), SI, facilitating conditions (FC), IC, culture, and mobile technology (MT). This led to the development of hypotheses 1a to 1i:

- Hypothesis 1a: Environmental aspects influence PE toward patients’ use of mHealth for self-monitoring (mHealthSM) for diabetes.
- Hypothesis 1b: Environmental aspects influence EE toward patients’ use of mHealthSM for diabetes.
- Hypothesis 1c: Environmental aspects influence SI toward patients’ use of mHealthSM for diabetes.
- Hypothesis 1d: Environmental aspects influence FC toward patients’ use of mHealthSM for diabetes.
- Hypothesis 1e: Environmental aspects influence IC of attitude toward patients’ use of mHealthSM for diabetes.
- Hypothesis 1f: Environmental aspects influence IC of beliefs toward patients’ use of mHealthSM for diabetes.
- Hypothesis 1g: Environmental aspects influence IC of skills toward patients’ use of mHealthSM for diabetes.
- Hypothesis 1h: Environmental aspects influence culture toward patients’ use of mHealthSM for diabetes.
- Hypothesis 1i: Environmental aspects influence MT toward patients’ use of mHealthSM for diabetes.

#### PE Hypothesis

PE, also known as perceived usefulness in the technology acceptance model or relative advantage in the diffusion of innovation, refers to the degree to which patients believe that self-monitoring mHealth systems will enable them to adhere effectively and efficiently to medication prescriptions [29,30,39]. In terms of this construct, usefulness in patients’ daily lives is included along with the likelihood that patients will be able to manage their lives successfully; thus, adherence to medication prescriptions will be augmented. The study found that patient self-monitoring capabilities were enhanced by gender, age, and experience in the use of mHealth systems. This understanding led to the development of hypothesis 2:

- Hypothesis 2: PE influences BI toward patients’ use of mHealthSM for diabetes.

#### EE Hypothesis

As with any other technology, mHealthSM may be perceived as either an easy or difficult tool to apply. Hence, EE can be understood as the degree to which patients perceive mHealthSM as either easy to use or requiring no effort to use [29,30,39]. This construct has attributes that include the ease of use of the mHealth system, clarity and understanding, and skill with respect to using the mHealth self-monitoring system. The influence of EE on the use of mHealthSM was moderated by gender, age, and experience. The hypothesis 3 was developed based on EE:

- Hypothesis 3: EE influences BI toward patients’ use of mHealthSM for diabetes.

#### SI Hypothesis

As the term implies, SI refers to a set of norms and values shared by a group of like-minded people [30,31,38,39]. Attributes that fall under this construct include proximity to other influential people and interest and willingness to learn from others’ experiences. It has been proposed that gender and age moderate SIs. Hence, the hypothesis 4 was developed based on this construct:

- Hypothesis 4: SI influences BI toward patients’ use of mHealthSM for diabetes.

#### FC Hypothesis

This refers to the support patients expect from health care providers. Such support might include raising awareness about mHealth, training in its use, and improving the availability of services [30,39]. Among the attributes of this construct are the availability of health care resources, including human and medical resources, health information, training, and financial and technical assistance. Using this construct, the hypothesis 5 was created:

- Hypothesis 5: FC influence BI toward patients’ use of mHealthSM for diabetes.

#### IC Hypothesis

IC refers to the nontechnical factors possessed by an individual that enable him or her to participate in the adoption and use of technological innovations [31]. People’s characteristics are largely influenced by their cultural perspectives, beliefs, educational and sociotechnical backgrounds, and socioeconomic status. In this study, this construct encompasses attributes associated with attitudes toward mHealth. Furthermore, it includes patients’ beliefs, skills in using MT, and trust that allows them to use mHealth self-monitoring systems. Based on this understanding, hypotheses 6a-c were developed:

- Hypothesis 6a: IC of attitude influence BI toward patients’ use of mHealthSM for diabetes.
- Hypothesis 6b: IC of beliefs influence BIs toward patients’ use of mHealthSM for diabetes.
- Hypothesis 6c: IC of skills influence BI toward patients’ use of mHealthSM for diabetes.

#### Culture Hypothesis

In the context of this study, culture consists of diverse aspects, including a patient’s community, its values and norms, and its traditional or collective attitudes toward technology. The SI concept also includes direct actions that affect the life and work of an individual [40]. As a result of this construct, the hypothesis 7 was developed:

- Hypothesis 7: Culture influences BI toward patients’ use of mHealthSM for diabetes.

#### MT Hypothesis

mHealth self-monitoring systems integrate mobile computing, medical sensors, and communication technologies. Goldfine et al [41] highlighted numerous wireless technologies that can be used to track a patient’s health. Wearable sensors are among the most widely used devices. Self-monitoring with these sensors increases accessibility, provides continuous feedback, and remains relatively noninvasive while contributing significantly to self-monitoring. The MT construct incorporates aspects of complexity, compatibility, scalability, motivation, and persuasiveness. A hypothesis 8 was developed from this construct:

- Hypothesis 8: MT influences BI toward patients’ use of mHealthSM for diabetes.

#### BI Hypothesis

It is essential that health care interventions are categorized correctly and correlated with behavioral analysis so that patients are empowered to self-monitor their health [25,42]. Technology acceptance and use theories also concur that the intention to act must be formed for a behavior to occur [26,27,29,30]. Therefore, BI plays an important role in predicting actual behavior. Most theories and models developed to inform technology acceptance and use are based on the assumption that an individual’s actual behavior is preceded by positive intentions [30,31,38,39]. For patients to use the mHealth self-monitoring system, they must first develop the intention to do so [26,27]. Therefore, hypothesis 9 was developed:

- Hypothesis 9: BI has a direct positive influence on patients’ use of mHealthSM for diabetes.

### Interacting Effects of Moderating Factors

#### Overview

Four moderating factors were identified and included in this study’s conceptual model. These included age, gender, experience, and motivation. The following interacting effects were predicted for each moderating factors.

#### Age

This study predicted that younger users tend to use technological innovations more readily than older users. Several studies have found that age moderates the acceptance, adoption, and use of technological innovations [30,31,39,43]. This study suggests that age could influence BIs via PE, EE, SI, IC, and culture. This led to hypothesis 10 being proposed. Subhypotheses for each construct were developed to examine the interacting effects of age on mHealthSM use:

- Hypothesis 10: Patient age has a moderating effect on the influence of PE, EE, SI, IC, culture, and MT toward BI, such that the moderating effects are higher for younger than older patients.

#### Gender

Various studies have proposed that gender can moderate the effects of independent variables and BIs on the actual behavior of acceptance, adoption, and use of technology [30,39,43]. This study predicted that gender would moderate the influence of PE, EE, SI, IC, culture, and MT on BI to inform patients of their use of mHealthSM. Therefore, the hypothesis 11 was developed:

- Hypothesis 11: The patient’s gender has a moderating effect on the influence of PE, EE, SI, IC, culture, and MT on BI, such that the moderating effect is higher in men than women.

#### Experience

Studies by Venkatesh et al [30,39] posited that individuals with experience find it easier to use new technological innovations than those with little or no experience. Thus, experience was proposed to have interacting effects on the influence of PE, EE, IC, MT, and FC on mHealthSM use by patients. This leads to the development of the hypothesis 12:

- Hypothesis 12: The patient’s experience has a moderating effect on the influence of PE, EE, FC, IC, culture, and MT on BI and that of BI toward mHealthSM use, such that the moderating effects are higher in patients with experience than those without.

#### Motivation

There is evidence that both intrinsic and extrinsic motivations have strong interacting effects on BI and actual technology use [27,39,44]. Alternatively, motivation has been seen as a moderating factor that could speed up the influence of one variable on BI and behavior. Motivation was adopted as a moderating factor with interacting effects on IC and BI, resulting in the hypothesis 13:

- Hypothesis 13: Motivation has a moderating effect on the influence of IC on BI and that of BI on mHealthSM use. The moderating effects appeared to be higher in patients who were highly motivated than those with low motivation.

## Methods

### Overview

Data for this study were collected from district hospitals in South Africa. Indicator trends for districts and provinces in the 2018-2019 District Health Barometer provided a snapshot of health care services in the public sector of South Africa, including the prevalence of common disease burdens and health care delivery [45]. On the basis of the population of each province and district municipality, this report presents estimates of the prevalence of diabetes. This revealed wide differences between provinces in diabetes prevalence and treatment coverage. According to a report, in 2017, diabetes among adults aged ≥15 years was more prevalent in the Western Cape, KwaZulu-Natal, Eastern Cape, Free State, Northern Cape, Gauteng, Limpopo, Northwest, and Mpumalanga provinces. In contrast, treatment rates were higher in the Northern Cape, Western Cape, Free State, Limpopo, KwaZulu-Natal, Eastern Cape, Gauteng, Mpumalanga, and Northwest regions [45]. On the basis of this report, geographic locations for selecting the study population were identified.

### Population and Sampling

Three conditions were considered in the selection of the study population. These were selecting provinces with high prevalence but low treatment rates, provinces with high prevalence and high treatment rates, as well as provinces within a good proximity but with a relatively dense population and urbanization. On the basis of these 3 conditions, the provinces of KwaZulu-Natal, the Western Cape, as well as Gauteng and Mpumalanga were selected. However, this study opted for district hospitals; there is only 1 regional hospital in each province that normally acts as a referral. This was expected to make data collection somewhat slower because of the busy schedules of health care providers in these regional hospitals.

Aspects of privacy, confidentiality, and avoidance of stigmatization of patients led to the provincial ethical clearance committees, from which authorization was sought for data collection, deeming it inappropriate to collect data from patients. However, because the aim of this study was to model the behavior of patients with diabetes regarding medication adherence, using health care providers was sufficient for the study. This study acknowledges that personal traits and characteristics play a significant role in determining an individual’s behavior. However, medical personnel and health workers can effectively and efficiently monitor patients’ daily routines and observe any deviations. In addition, health care providers can recognize patients’ functional and cognitive decline in their daily lives [46]. This confirmed the eligibility of health care providers as respondents of the study. Therefore, only health care providers, such as physicians and social workers, who are regularly involved in counseling, medication prescriptions, and treatment of patients with diabetes, were included in the study.

The pre-exploratory study that had been conducted earlier revealed that many South African district hospitals do not have specific diabetic clinics and that patients with diabetes are treated in exactly the same way as other patients in general wards or outpatient clinics. This implies that all medical personnel and social workers were qualified to form the study population. These staff members regularly dealt with patients with diabetes. Information also revealed that there were 30 to 50 medical personnel and social workers at a district municipality hospital. On the basis of the number of district hospitals in the 4 sampled provinces, the population of the study was 660, giving a sample size of 248 respondents when using the Krejcie and Morgan [47] tool for determining the sample size of a finite population. Simple random sampling was then applied based on the inclusive criterion that a respondent should be either a medical personnel or a health worker.

On the basis of the sample size, 350 questionnaires were distributed, of which 257 were returned, giving a response rate of 73%. This was deemed sufficient for the analysis. This study set out to analyze the moderating effects of the demographic and situational variables, thus variance-based structural equation modeling using smart partial least squares was applicable for data analysis.

### Ethical Considerations

This study was conducted as part of a doctoral study at the University of South Africa. It is mandatory that all studies conducted within the institution receive ethical approval before the commencement of data collection, as no retrospective approval could be allowed. Therefore, this study was approved by the University of South Africa Instructional Ethics Board. Moreover, collecting any medical-related data, whether from patients or health care providers in South Africa, requires the researcher to register the research in the medical database and to receive ethics clearance from each province to participate in the study. The main researcher, who is the corresponding author, sought permission and scheduled presentations of the proposal at each of the 3 provinces’ regional health departments before ethical approval could be granted. This was done, and ethical approval was received only to collect data from health care providers. The questionnaire for data collection was accompanied by a cover letter, which the respondents of the study were reminded to read before filling the questionnaire. The letter explained to the respondents about their privacy and confidentiality protection, their rights, and freedom to drop out of the study at any time without such decision prejudicing their rights. Most importantly, the respondents were informed that the data were intended for academic purposes only and that none of their particulars or those of their health institutions would be included anywhere in the study. Moreover, respondents were reminded that there were no monetary benefits or compensation expected from filling out the questionnaire.

## Results

### Overview

**Figure 2:**
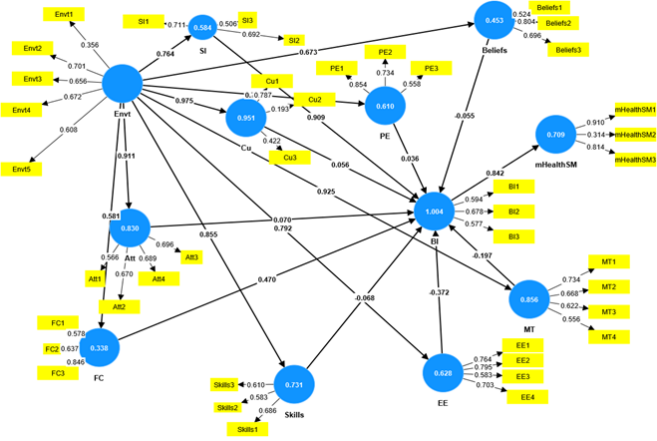
Measurement models of nonmoderated constructs. Att: Attitude; BI: Behavioral Intention; Cu: Culture; EE: Effort Expectancy; Envt: Environment; FC: Facilitating Conditions; mHealthSM: Mobile Health Self-monitoring; MT: Mobile Technology; PE: Performance Expectancy; SI: Social Influence.

The collected data were screened and cleaned, and outliers were eliminated, leaving 158 samples in the final data sets. Two measurement models were built with both moderated and nonmoderated constructs. These measurement models were evaluated, modified, and adjusted to form the path models. Figure 2 shows the nonmoderated measurement model of the constructs.

From the measurement model in Figure 2, the correlated values between the observed variables and the latent variables, the *R*^2^ of the model, and the coefficient of the linear path regression between the latent variables were determined. Figure 2 also provided results for the convergent validities obtained by observing the average variance extracted (AVE), the internal consistency values (Cronbach α) and the composite reliability (CR; Dillon-Goldstein þ—rho) as demonstrated in Table 1.

**Table 1:** Construct reliability and validity.

	Cronbach α	Composite reliability (rho_a)	Composite reliability (rho_c)	Average
Attitude	.754	0.756	0.751	0.432
Behavioral intention	.640	0.653	0.649	0.382
Beliefs	.710	0.745	0.720	0.469
Culture	.407	0.626	0.476	0.279
Effort expectancy	.806	0.816	0.806	0.512
FC^a^	.726	0.760	0.733	0.485
MT^b^	.738	0.749	0.741	0.420
Performance expectancy	.762	0.791	0.764	0.527
Skills	.667	0.664	0.660	0.394
Social influence	.672	0.691	0.675	0.414
Environmental factors	.726	0.763	0.741	0.374
mHealthSM^c^	.752	0.847	0.747	0.530

^a^FC: facilitating conditions.

^b^MT: mobile technology.

^c^mHealthSM: mHealth for self-monitoring.

CR prioritizes variables according to their reliability and is considered the most appropriate for partial least squares. In contrast, Cronbach α, which measures internal consistency, is sensitive to the number of variables in a construct [48]. CR and Cronbach α were used to determine whether the study sample was biased. Thus, values between .60 and .70 should be noted, as well as .70 to .90, respectively, to achieve good fit and satisfactory results [49]. However, as shown in Table 1, the cultural construct had an AVE of 0. 279 (<0.50), Cronbach α of .407 (<.6), and CR values of less than 0.7. These results suggest that either items linked to culture should be eliminated during adjustments and modification of the model or that the construct should not be included for further analysis. Because culture had only 3 measurement items and was moderated by gender and age, further analysis of the construct is needed. The moderating effects had to be evaluated to determine whether they affected the prediction. Table 1 further shows that most constructs had AVEs less than 0.5, indicating that model modifications were necessary.

Figure 2 was also used to compute discriminant validity, which measures the independence of one construct from the other. In this study, the Heterotrait-Monotrait ratio (HTMT) of correlations was applied [49]. The results are summarized in Table 2. Fornell and Larcker [50] indicated that values for HTMT not exceeding 0.9 could be accepted, although HTMT values near 1 indicated a lack of discriminant validity. If the value of the HTMT is higher than the threshold, then there is a lack of discriminant validity, which suggests that the model should be adjusted.

**Table 2:** Heterotrait-monotrait ratio (HTMT) list.

	HTMT
Behavioral intention→attitude	0.479
Beliefs→attitude	0.806
Beliefs→behavioral intention	0.675
Culture→attitude	1243
Culture→behavioral intention	0.690
Culture→beliefs	1.019
Effort expectancy→attitude	0.882
Effort expectancy→behavioral intention	0.581
Effort expectancy→beliefs	0.664
Effort expectancy→culture	0.924
FC^a^→attitude	0.570
FC→behavioral intention	0.964
FC→beliefs	0.744
FC→culture	0.776
FC→effort expectancy	0.537
MT^b^→attitude	0.766
MT→behavioral intention	0.351
MT→beliefs	0.674
MT→culture	0.949
MT→effort expectancy	0.670
MT→FC	0.468
PE^c^→attitude	0.707
PE→behavioral intention	0.675
PE→beliefs	0.608
PE→culture	0.891
PE→effort expectancy	0.794
PE→FC	0.550
PE→MT	0.567
Skills→attitude	0.933
Skills→behavioral intention	0.738
Skills→beliefs	0.931
Skills→culture	1.005
Skills→effort expectancy	0.909
Skills→FC	0.755
Skills→MT	0.772
Skills→PE	0.949
Social influence→attitude	0.699
Social influence→behavioral intention	0.909
Social influence→beliefs	0.793
Social influence→culture	0.980
Social influence→effort expectancy	0.845
Social influence→FC	0.909
Social influence→MT	0.597
Social influence→PE	0.864
Social influence→skills	0.911
Environmental factors→attitude	0.941
Environmental factors→behavioral intention	0.545
Environmental factors→beliefs	0.681
Environmental factors→culture	1.165
Environmental factors→effort expectancy	0.800
Environmental factors→FC	0.600
Environmental factors→MT	0.953
Environmental factors→PE	0.785
Environmental factors→skills	0.865
Environmental factors→social influence	0.760
mHealthSM^d^→attitude	0.613
mHealthSM→behavioral intention	0.824
mHealthSM→culture	0.783
mHealthSM→effort expectancy	0.622
mHealthSM→FC	0.680
mHealthSM→MT	0.457
mHealthSM→PE	0.596
mHealthSM→skills	0.682
mHealthSM→social influence	0.784
mHealthSM→environmental factors	0.580

^a^FC: facilitating conditions.

^b^MT: mobile technology.

^c^PE: performance expectancy.

^d^mHealthSM: mHealth for self-monitoring.

The results in Table 2 indicate that culture and skills constructs do not exhibit good discriminant validity. However, most constructs had acceptable discriminant validity values that were below the threshold. The results confirmed the need to adjust the model and exclude cultures from the final model. Observed and latent variables were also checked for cross-loadings. It is recommended that during cross-loading, an item’s component loading on its own construct should be higher than that on other constructs. Researchers such as Ringle et al [49] and Henseler et al [51] recommended that when an item loads higher onto a construct other than its parent construct, the discriminant validity is compromised. A difference between loads less than 0.10 indicates higher cross-loading onto the other construct, which compromises discriminant validity. With the exception of culture, skills, and environment constructs, most variables had higher cross-loadings on their parent constructs than on the other constructs. Again, this indicates that modifications to the model were necessary. As this study tested for the direct influence of constructs and for the influence when the relationship of the construct is moderated, constructs whose HTMT was beyond the threshold were not discarded, as there was a need to check if their influencing effects would change with the moderating effects.

### Modification of the Measurement Models

Variance-based structural equation modeling (SEM) has no specific fit indices tested, unlike other measures; therefore, covariance-based SEM may be used [52]. Among these is the Bentler-Bonett Normed Fit Index, an incremental measure of goodness of fit that has not been affected by the number of variables in the model for fitness (Normed Fit Index >0.90). In addition, the root mean square residual threshold is 0.08; the exact fit measures the squared Euclidean distance and the geodesic distance, which should have a nonsignificant probability (*P*>.05); and there is the chi-square value (×2) [49,52]. Table 3 shows the fitness of the model.

**Table 3:** Model fit summary.

	Saturated model	Estimated model
SRMR^a^	0.074	0.079
d_ULS^b^	6017	10,323
d_G^c^	2355	2740
*χ*^2^ (*df*)	1,643,684	1,860,735
NFI^d^	0.906	0.954

^a^SRMR: standardized root mean square residual.

^b^bd_ULS: the squared Euclidean distance.

^c^d_G: the geodesic distance.

^d^NFI: Normed Fit Index.

### Path Model and Significance of Independent Variables

After fitting the measurement model, a path model was constructed and used to determine the significance of the contribution of each construct. The Bootstrap result approximates the normality of the data for a 2-tailed test at different confidence levels. For a 95% CI, significance was obtained when T value was 1.96 at the .05 level [49,52]. Table 4 presents the significance of the independent variables.

**Table 4:** Means, SDs, T statistics, and *P* values of the independent variables.

	Original sample (O)	Sample, mean (SD)	T statistics (|O/SD|)	*P* values
Attitude→behavioral intention	0.703	0.419 (0.076)	9.280	.01
Behavioral intention→mHealthSM^a^	0.842	0.850 (0.074)	11.377	<.001
Beliefs→behavioral intention	−0.554	−0.290 (0.069)	8.001	.02
Culture→behavioral intention	0.056	1318 (40.526)	0.001	.99
Effort expectancy→behavioral intention	−0.372	−0.561 (0.072)	5.203	.03
FC^b^→behavioral intention	0.470	0.427 (0.078)	6.021	.004
MT^c^→behavioral intention	−0.197	−0.302 (0.096)	2.043	.04
Performance expectancy→behavioral intention	0.636	0.891 (0.209)	3.043	.02
Skills→behavioral intention	−0.068	0.920 (63.387)	0.001	.99
Social influence→behavioral intention	0.909	−0.291 (65.091)	0.014	.99
Environmental factors→attitude	0.911	0.907 (0.066)	13.829	<.001
Environmental factors→beliefs	0.673	0.682 (0.095)	7.078	<.001
Environmental factors→culture	0.975	0.991 (0.093)	10.477	<.001
Environmental factors→effort expectancy	0.792	0.791 (0.064)	12.427	<.001
Environmental factors→FC	0.581	0.589 (0.139)	4.169	<.001
Environmental factors→MT	0.925	0.923 (0.071)	13.049	<.001
Environmental factors→performance expectancy	0.781	0.785 (0.070)	11.097	<.001
Environmental factors→skills	0.855	0.877 (0.112)	7.639	<.001
Environmental factors→social influence	0.764	0.777 (0.075)	10.203	<.001

^a^mHealthSM: mHealth for self-monitoring.

^b^FC: facilitating conditions.

^c^MT: mobile technology.

As shown in Table 4, the 3 independent variables—culture, skills, and SI—contributed to the overall prediction of the model as being not significant. On the basis of Figure 1, it was theorized that a patient’s environment influences the use of mHealthSM. This theory was confirmed by the results of the study, which showed that the influence of environmental aspects was significant for all independent variables (all *P*<.001).

### Testing of the Hypotheses

Using the T statistics provided in Table 4, the suggested hypotheses are tested at a level of .05. The results are as shown in Table 5.

**Table 5:** Hypotheses testing.

Hypotheses	T statistic	*P* values	Comment
Hypothesis 1a: environmental aspects influence performance expectancy toward patients’ use of mHealthSM^a^ for diabetes.	11.097	<.001^b^	Accepted
Hypothesis 1b: environmental aspects influence effort expectancy toward patients’ use of mHealthSM for diabetes.	12.427	<.001^b^	Accepted
Hypothesis 1c: environmental aspects influence social influence toward patients’ use of mHealthSM for diabetes.	10.203	<.001^b^	Accepted
Hypothesis 1d: environmental aspects influence facilitating conditions toward patients’ use of mHealthSM for diabetes.	4.169	<.001^b^	Accepted
Hypothesis 1e: environmental aspects influence individual characteristics of attitude toward patients’ use of mHealthSM for diabetes.	13.829	<.001^b^	Accepted
Hypothesis 1f: environmental aspects influence individual characteristics of beliefs toward patients’ use of mHealth in self-monitoring for diabetes.	7.078	<.001^b^	Accepted
Hypothesis 1g: environmental aspects influence individual characteristics of skills toward patients’ use of mHealthSM for diabetes,	7.639	<.001^b^	Accepted
Hypothesis 1h: environmental aspects influence culture toward patients’ use of mHealthSM for diabetes.	10.477	<.001^b^	Accepted
Hypothesis 1i: environmental aspects influence mobile technology toward patients’ use of mHealthSM for diabetes.	13.049	<.001^b^	Accepted
Hypothesis 2: performance expectancy influences behavioral intention toward patients’ use of mHealthSM for diabetes.	3.043	.02^b^	Accepted
Hypothesis 3: effort expectancy influences behavioral intention toward patients’ use of mHealthSM for diabetes.	5.203	.03^b^	Accepted
Hypothesis 4: social influence influences behavioral intention toward patients’ use of mHealthSM for diabetes.	0.014	.99^c^	Rejected
Hypothesis 5: facilitating conditions influence behavioral intention toward patients’ use of mHealthSM for diabetes,	6.021	.004^b^	Accepted
Hypothesis 6a: individual characteristics of attitude influence behavioral intentions toward patients’ use of mHealthSM for diabetes.	9.280	.01^b^	Accepted
Hypothesis 6b: individual characteristics of beliefs influence behavioral intention toward patients’ use of mHealthSM for diabetes.	8.001	.02^b^	Accepted
Hypothesis 6c: individual characteristics of skills influence behavioral intention toward patients’ use of mHealthSM for diabetes.	0.001	.99^c^	Rejected
Hypothesis 7: culture influences behavioral intention toward patients’ use of mHealthSM for diabetes.	0.001	.99^c^	Rejected
Hypothesis 8: mobile technologies influence behavioral intention toward patients’ use of mHealthSM for diabetes.	2.043	.04^b^	Accepted
Hypothesis 9: behavioral intention has a direct positive influence on patients’ use of mHealthSM for diabetes.	11.377	<.001^b^	Accepted

^a^mHealthSM: mHealth for self-monitoring.

^b^*P*<.05.

^c^*P*>.05.

The results from Table 5 indicate the reasons for all hypotheses except culture, IC of skills, and SI being accepted.

### Analysis of the Moderating Variables

Four moderating variables—age, gender, experience, and motivation—were tested for their interactions. To read the adjusted coefficients, the structural model was redesigned, the model quality was determined, adjustments were made, and a path analysis was conducted to determine the moderating coefficients. Multimedia Appendix 1 presents the results.

The suggested hypotheses were tested by comparing the significance of the interacting effects of the moderating factors based on the T statistic and the *P* value for each relation. When the significance of the interaction was observed, it implied that the hypothesized interaction existed, and when it was not observed, it implied that the interaction did not exist. Table 6 presents the tested hypotheses.

**Table 6:** Moderating factors hypotheses testing.

Moderating factor: hypothesis		T statistic	*P* values	Comment
**Age—hypothesis 10: the patient’s age has a moderating effect on the influence of PE^a^, EE^b^, SI^c^, IC^d^, Cu^e^, and MT^f^ toward BI^g^, such that the moderating effects are higher in younger than older patients.**				
	Age × PE→BI	0.443	.66^h^	Rejected
	Age × EE→BI	1.969	.04^i^	Accepted
	Age × SI→BI	0.689	.49^h^	Rejected
	Age × attitude→BI	4.925	.003^i^	Accepted
	Age × beliefs→BI	3.458	.03^i^	Accepted
	Age × skills→BI	0.416	.68^h^	Rejected
	Age × Cu→BI	2.128	.03^i^	Accepted
	Age × MT→BI	1.367	.17^h^	Rejected
**Gender—hypothesis 11: the patient’s gender has a moderating effect on the influence of PE, EE, SI, IC, Cu, and MT for BI, such that the moderating effects are higher in men than women.**				
	Gender × PE→BI	1.559	.12^h^	Rejected
	Gender × EE→BI	3.473	.001^i^	Accepted
	Gender × SI→BI	2.660	.008^i^	Accepted
	Gender × attitude→BI	5.112	.02^i^	Accepted
	Gender × beliefs→BI	7.608	<.001^i^	Accepted
	Gender × skills→BI	1.477	.08^h^	Rejected
	Gender × Cu→BI	1.324	.19^h^	Rejected
	Gender × MT→BI	0.911	.36^h^	Rejected
**Experience—hypothesis 12: the patient’s experience has a moderating effect on the influence of PE, EE, FC, Cu, and MT toward BI and that of BI to mHealthSM^j^ use, such that the moderating effects are higher in patients with experience than those without.**				
	Experience × PE→BI	0.410	.68^h^	Rejected
	Experience × EE→BI	2.572	.02^i^	Accepted
	Experience × FC→BI	0.372	.71^h^	Rejected
	Experience × Cu→BI	2.154	.02^i^	Accepted
	Experience × MT→BI	1.249	.21^h^	Rejected
	Experience × BI→mHealthSM	7.758	<.001^i^	Accepted
**Motivation—hypothesis 13: motivation has a moderating effect on the influence of IC toward BI and that of BI toward mHealthSM use, such that the moderating effects are higher in highly motivated patients than those with low motivation.**				
	Motivation × attitude→BI	6.367	.002^i^	Accepted
	Motivation × skills→BI	1.017	.47^h^	Rejected
	Motivation × beliefs→BI	5.003	.01^i^	Accepted
	Motivation × BI→mHealthSM	4.739	<.001^i^	Accepted

^a^PE: performance expectancy.

^b^EE: effort expectancy.

^c^SI: social influence.

^d^IC: individual characteristics.

^e^Cu: culture.

^f^MT: mobile technology.

^g^BI: behavior intention.

^h^*P*>.05.

^i^*P*<.05.

^j^mHealthSM: mHealth for self-monitoring.

## Discussion

### Interpretation of Findings

#### Overview

Over the past few decades, there has been a growing trend toward computerizing health care with the hope of improving health outcomes, reducing costs for both health care providers and patients, and improving the ease of access to data and sharing of health care information [12]. On the same note, experiences during the COVID-19 pandemic proved that technological innovations should be effectively and efficiently used for health care and the number of patients visiting doctors will drop exponentially, which will reduce the weight placed on the health care systems of countries [53]. Patients’ self-monitoring of their health requires behaviors and positive lifestyle changes to adhere to medical prescriptions. This study was based on using behavioral change aspects to develop a model that could be used as a guideline for the implementation of an mHealth self-monitoring system. Based on the conceptual model underpinned by extended version of the UTAUT, this study theorized 18 hypotheses from the constructs of the model and 4 major hypotheses from the moderating factors (age, gender, experience, and motivation). The age-, gender-, and experience-moderating factors each had 6 subhypotheses, whereas motivation had 2 hypothesized interacting effects. The results of the study revealed that environmental aspects, PE, EE, FC, IC of attitude and beliefs, and MT factors are significant for patients’ BI to use an mHealth self-monitoring system. The other factors of SI and culture were found to be significant only after being moderated by gender and age. This section discusses the findings, as shown in Tables 4-6, and provides interpretations based on theory and practice.

#### Environmental Aspects Findings

All hypotheses related to this construct were accepted. The implication of this finding is that the availability of health care workers correlates directly with the health care provision, the number of health care providers, their skill level, and where and how they are deployed and managed. In resource-limited countries, there is a skewed distribution of experienced and highly qualified medical personnel in urban areas compared with rural settings [1,54]. Rural settings are typically disadvantaged by such skewedness when it comes to health care personnel, which calls for mHealth self-monitoring. Furthermore, the migration of health care personnel to rural settings due to increased urbanization has exacerbated access and equity issues in the provision of health care, as well as the monitoring of patients with chronic conditions in rural settings [3,4]. Further evidence of the influence of environmental factors was observed during the COVID-19 pandemic lockdown, in which mortality rates for chronically ill patients increased because of poor accessibility to medical facilities [53,55].

#### PE Findings

The proposed hypothesis based on the PE construct was accepted. These findings indicate that PE, also known as expected benefits, is essential for patients’ expectations of mHealth self-monitoring. Patients expect such benefits from taking care of their lives. Additionally, the mHealth self-monitoring system can be perceived by patients as a tool to address health inequities. Such inequalities arise from limited access to health facilities, unmet needs due to poverty, and government failure to provide services to underserved communities. These findings concur with those of Kalema and Musoma [1], Venkatesh et al [39], Momani [31], and Narsai et al [54], who indicated that patients’ perception of expected benefits from technology is essential and a key contributing factor to their actual use of technology.

#### EE Findings

This theorized relationship was accepted in light of this construct. EE, which in this study is considered the ease of use of the mHealth self-monitoring system, has been found to be significant in many studies. This is because the users’ perception is that once the technology is easy to use, it will benefit them [32]. In addition, when patients find the mHealth self-monitoring system easy to use, they gain trust in it, which leads to a high level of self-efficacy, all of which is necessary for actual use.

In relation to this study, EE may also be explained by the ability of patients to own a mobile phone and to read, interpret, and comprehend SMS text messages sent as reminders for adherence to medication. As much as the exponential growth of mobile telephones in resource-limited countries makes smartphones available and accessible, understanding and interpreting the SMS text messages sent by the system is paramount for daily medicine adherence. These findings agree with those of researchers such as Islam et al [17] and Chifu et al [46], who noted that patients’ adherence to daily routine medicine prescriptions induces calm and comfort, allaying anxiety and stress. Moreover, independence in monitoring one’s life is fundamental to improving quality of life. This could help patients with chronic diseases live a long, meaningful, and dignified life.

#### SI Findings

The suggested hypothesis for the SI construct was supported. The SI on perceptions may not carry much weight. Rather than accepting the influence of others, a patient will decide to use the system after perceiving it as beneficial to his or her life. However, when looking at the support patients gain from the communities within their environment, such as information provision, interpersonal contacts, and health groups, it may have a strong influence on their BI. This also explains why SI was significant when moderated by gender, as shown in Table 6.

With regard to chronic diseases, regarding patients’ adherence to medical prescriptions, SI presents a split understanding: some patients may prefer to keep their illness private. In this case, SI may not be considered a contributing factor to the use of mHealth self-monitoring systems [56]. These findings do not support those of Cruz-Ramos et al [38], Woldeyohannes and Ngwenyama [57], and Kruse et al [58], who indicated that SI is an essential antecedent to mHealth use. However, this study also acknowledges that as some patients may be unfamiliar with new technological applications, such as the mHealth app, where SI could play a significant role.

#### FC Findings

It was hypothesized that the support provided by health facilities, governments, and health care personnel would influence patients’ intention to use mHealth self-monitoring systems. Thus, this hypothesis was accepted. Facilitation can take the form of financing; the provision of services; stewardship; and resource development, including human resources, physical infrastructure, and knowledge sharing [56]. Consequently, patient satisfaction with self-monitoring systems through mHealth is dependent on the availability of technical infrastructure, such as mobile networks and bandwidth strength, the ability of health workers to reach rural communities, and the availability of health personnel support. A number of researchers have also argued that the successful implementation of health systems depends on the support health institutions receive in terms of better policies, standards, and strategies, as well as their evaluation and reform [1,8,59].

#### IC Findings

This study hypothesized IC based on 3 categories: attitudes toward mHealth self-monitoring systems, beliefs regarding mHealth use, and the skills needed to use them. Both the attitude and belief hypotheses were accepted, whereas the skills hypothesis was rejected. These findings imply that when patients are aware that the use of mHealth to keep track of their own health will ameliorate their lives, they will develop better attitudes toward using the system, concomitantly with a strong belief that their lives will improve. In contrast, once patients establish a positive attitude and a strong belief, they do not need extra skills to monitor their health. This study agrees with the beliefs of Islam et al [17], Chatterjee [21], and Wu et al [56], who indicated that mHealth empowers patients to manage their health efficiently and effectively, thereby enabling them to develop positive attitudes, satisfaction, and beliefs that they are on track in ameliorating their lives.

#### Culture Findings

A hypothesis was drawn on the influence of culture on patients’ intentions to use mHealth for self-monitoring of their health; however, this hypothesis was rejected. The implications of the findings are that, despite the fact that culture has been found to be influential in some technology acceptance and use studies, its influence on self-management of one’s health may not be noteworthy [60,61]. Culture is, in most cases, considered a significant factor in addressing inequalities and inequities in health. Culture is also essential for closing the gaps in the social determinants of health. However, inequalities and inequities may not apply in the case of mHealth self-monitoring systems. This is because a system has already been developed to assist people, regardless of their social status. The findings of this study disagree with those of previous researchers such as Chatterjee [21] and Pourmand et al [62], who found culture to be a significant factor in the use of mHealth. In contrast, this study’s findings agree with those of Asmah et al [63], who argued that well-sensitized patients with chronic disease will use mHealth without cultural bias and misconceptions of the causes of the diseases and with no fear of treatment.

#### MT Findings

In this construct, we examined how MT influences patients’ intention to use mHealth self-monitoring systems. Thus, this hypothesis was accepted. The acceptance of this hypothesis implies that mobile technologies and their associated attributes are crucial for self-monitoring. These findings agree with those of Kalema and Musoma [1], Istepanian and Al-Anzi [61], and Kruse et al [64], who have also suggested that MT is the cornerstone of self-management systems due to its pervasiveness. The results of this study are also consistent with those of Goldfine et al [41], who indicated that wearable technologies can enhance the significance of mHealth by continuously monitoring patients’ drug use, particularly for patients with chronic disease who regularly take medicine.

#### BI Findings

The patients’ BIs were hypothesized to directly influence their use of mHealth self-monitoring systems. Thus, this hypothesis was accepted. BI has been found to be a significant mediating factor and a major antecedent of actual use in many studies on the adoption, acceptance, and use of technology [31,32,39]. These findings imply that individuals must first make a behavioral change to perform that particular act to decide whether or not to use a technology. The findings of this study are in line with those of many researchers such as David et al [29], Venkatesh et al [30], Momani [31], and Cruz-Ramos et al [38], who concurred with the studies by Fishbein and Ajzen [26,27], emphasizing the importance of BI in enlisting actual behavior.

### Findings in Relation to Moderating Factors

This section discusses and interprets the findings of the moderating factors hypotheses.

#### Age

Hypothesis 10 posited that patient age has a moderating effect on the influence of PE, EE, SI, IC, culture, and MT on BI, such that the moderating effects are higher in younger than older patients. The results indicated that moderating or interacting effects were observed for EE, IC of attitude, IC of beliefs, and culture. However, no interaction effects were observed with PE, SI, IC of skills, and MT. The implications of these findings are that there are instances in which the interacting effects of age become salient when using technology. As in the case of this study, younger patients would assimilate to the use of the mHealth self-monitoring system much more rapidly than older patients, leaving EE, attitude, and beliefs greatly impacted by the interacting effects of age. This finding also agrees with those of previous studies [65,66]. Similarly, young people may not have many cultural beliefs regarding the causes of chronic illnesses. Therefore, they are likely to seize the opportunity for any form of treatment presented to them.

#### Gender

Patients’ gender was hypothesized to have moderating effects on the influence of PE, EE, SI, IC, culture, and MT on BI, such that the moderating effects are higher in men than women. The results indicated that gender’s interacting effects exist with EE, SI, IC of attitude, and IC of beliefs. In contrast, gender’s interacting effects with PE, IC of skills, culture, and MT were found not to be significant, implying that there were moderating effects.

The implication of these findings is that in many instances, younger men will always be more ambitious in exploring technology, making them familiar with it, thus finding mHealth easier to use than in the case of their women counterparts. Similarly, younger men are more influenced by their peers than women, which stimulates their beliefs and attitudes toward technological innovation. The findings of this study are consistent with those of previous researchers [30,39,67,68], who also emphasized the influence of gender on attitudes and beliefs regarding the use of technology.

#### Experience

Patients’ experience with using mobile phones was hypothesized to have interacting effects on the influence of PE, EE, FC, culture, and MT on BI and that of BI toward mHealth self-monitoring use, such that the moderating effects are higher in patients with experience than those without. The results indicated that interaction effects of experience do exist with EE, culture, and BI, but not with PE, FC, and MT. In relation to this study, the implication of these findings is that experience is essential for mHealth self-monitoring system use. Patients with experience using mobile apps will find mHealth easier to use than their counterparts with no experience. The findings of this study agree with those of previous researchers [43,66,68], who indicated that when users have experience with technology, they will develop self-efficacy. This will help them use any other systems or upgrades with ease. In contrast, experience was found to have no interacting effects on FC and MT. This is because FC and MT are more external to the patients, with the former relating to health institutions while the latter is more related to the system architecture.

#### Motivation

This study hypothesized that motivation has moderating effects on the influence of IC on BI and that of BI on mHealth self-monitoring use. The moderating effects were greater in highly motivated patients than those with low motivation. The results indicated that the moderating effects of motivation exist with BI toward mHealth self-monitoring system use and the influence of IC of attitude and beliefs toward BI. However, this effect does not exist with the influence of IC of skills toward BI.

The implication of these findings is that motivation raises patients’ trust and willingness; as a result, they develop a positive attitude and beliefs toward using the mHealth system to monitor their health. The findings of this study are consistent with those of previous studies [39,44], which indicated that motivation is a major antecedent of behavioral change and, hence, essential for the actual performance of the behavior. Moreover, these findings agree with a previous study [24], which emphasized the role of motivation when using persuasive technology.

### Limitations and Recommendations

Patients’ management of their own lives is essential as this helps patients to remain healthy, minimizing their readmissions to hospitals, thereby reducing health-related costs and ameliorating their quality of life. With the possibility of future pandemics, it is no longer optional that health care should move toward real-time data analytics to improve timely decision-making related to saving lives. The pervasiveness of technological innovations in the health care domain to reduce the increasing disease burden has made mHealth a much sought-after tool to be leveraged in the health sector. mHealth systems not only work as a reminder system for patients but also as a source of information. Such systems allow health care professionals to collect quantitative information related to patients’ health and behavior regarding medication adherence. This helps health care professionals make meaningful decisions related to health risk prediction while developing more interventions.

Through the use of a close-ended questionnaire, this study gathered insights from diverse health care personnel interacting with patients with diabetes daily. The collected data were analyzed using smart partial least squares SEM to develop a model for mHealthSM. As demonstrated in this study, it could be deduced that with the ubiquitous adoption of smartphones across racial, educational, and socioeconomic groups, it is possible to develop new models of health care delivery for patients with chronic conditions, such as diabetes to promote health equality and equity. Therefore, it is vital that patients receive timely reminders to take their medicines at the right time and in the right quantity. This avoids double doses and enables prescriptions to be renewed over time. In addition, data collected from mHealth self-monitoring systems could be used to reduce clinical trial costs, thereby enabling pharmaceutical companies to gain accurate information about medicines, leading to the production of new drugs.

This study acknowledges that modeling individual behavior involves understanding perceptions. Hence, it would have been more helpful to use patients as respondents to report their own behaviors, rather than from health care providers. As much as health care providers monitor and can tell patients’ behavior toward medication adherence, there could be limitations in explaining why such nonadherence occurs. It is therefore recommended that future studies form joint research teams with health care personnel or medical experts, so the data are collected directly from patients rather than secondhand from health care providers.

This study was concerned with the development of a self-monitoring system for medication adherence only, and there was no intervention to influence health outcomes. However, there are also various other ways in which patients with diabetes may be monitored, such as the rate of physical activity, weight gain or loss, and blood glucose levels. These other health-monitoring facilities were outside the scope of this study. Future research could develop mHealth systems that combine all of these health conditions that should be monitored into one integrated system. Furthermore, owing to the increasing number of patients with chronic conditions, data storage and network stability may impede the effective use of the mHealth self-monitoring system. Therefore, this study recommends that future mHealth apps and device integration be developed to be supported by a more comprehensive, cloud-based system. Cloud-based solutions will provide various benefits, including stability, availability, and security, in addition to health care personnel now being in a position to analyze patient data from a central platform.

This study followed a quantitative approach in which respondents only answered questions with predetermined answers. Behavior patterns are influenced by a number of individual factors, many of which may not be expressed in words but by feelings and expressions. Such feelings and expressions can only be interpreted if the researcher has a one-to-one interaction with a participant in the form of qualitative interviews. Therefore, as much as the quantitative approach analyzes causal and interacting effects of moderating factors that are essential for determining individuals’ continuance behaviors, this study recommends that future research should use qualitative or mixed methods for the model’s validation.

### Contributions of the Study

This study contributes to the development of a model that incorporates the interacting effects of users’ moderating factors that have been recommended in various research studies on health interventions as being essential for the self-monitoring of patients. As recommended by other researchers, technological innovation may be accepted, adopted, and used; however, its continued use may not be sustained. Moderating factors are essential in predicting future use; the influence of some factors may cease, leading others to become salient. Hence, including moderating factors to predict health care interventions is a significant contribution, as the developed model will be used to extend research on health care interventions with confidence in predicting future maintenance use. Moreover, the developed model will be implemented in a fully functioning mHealth self-monitoring system that will be practically used by both health care providers and patients.

### Conclusions

The pervasiveness of the use of technological innovations in the health care domain and the increase in disease burden has made mHealth a sought-after tool in the health sectors of many countries, whether resource limited or high income. mHealth has been widely applied in different aspects of health care management, especially with chronic complications that require routine monitoring, making adherence a challenge [21]. The mHealth models, such as the one developed in this study, could be used to implement systems that not only work as a reminder system for patients but also allow health care professionals to collect quantitative information related to patients’ health and behavior toward medication adherence, which helps personnel to make meaningful decisions. Through the data generated, stored, and disseminated by mHealth systems, health care providers will be capable of gathering patients’ related data and making decisions, such as patients’ risk prediction, need for physical monitoring, or admission to intensive care.

Patients’ management of their own lives is essential, as this helps them to remain healthy and minimizes their readmissions to hospitals, thereby reducing health-related costs and improving their quality of life [21,46]. With the increasing world pandemics, it is no longer optional that health care moves toward real-time data analytics to improve timely decision-making related to saving lives. In an effort to do so, more technological interventions into health care and management are needed to remain abreast with the increasing globalization in the fourth industrial revolution era.

## Appendix

Multimedia Appendix 1 Mean, STDEV, T-values, and P-values of moderating variables.

## Abbreviations

AVE: average variance extracted
BI: behavioral intention
CR: composite reliability
EE: effort expectancy
FC: facilitating conditions
HTMT: Heterotrait-Monotrait ratio
IC: individual characteristics
mHealth: mobile health
mHealthSM: mobile health for self-monitoring
MT: mobile technology
PE: performance expectancy
SEM: structural equation modeling
SI: social influence
TDF: theoretical domains framework
UTAUT: Unified Theory of Acceptance and Use of Technology

## Data Availability

The data sets generated and analyzed during this study are available from the corresponding author upon reasonable request.
