# Supplementary figures and images for: mHealth Self-Monitoring Model for Medicine Adherence of Patients With Diabetes in Resource-Limited Countries: Structural Equation Modeling Approach
Source: JMIR Form Res. 2023 Oct 23;7:e49407. doi: 10.2196/49407 (PMC10628689; doi:10.2196/49407)

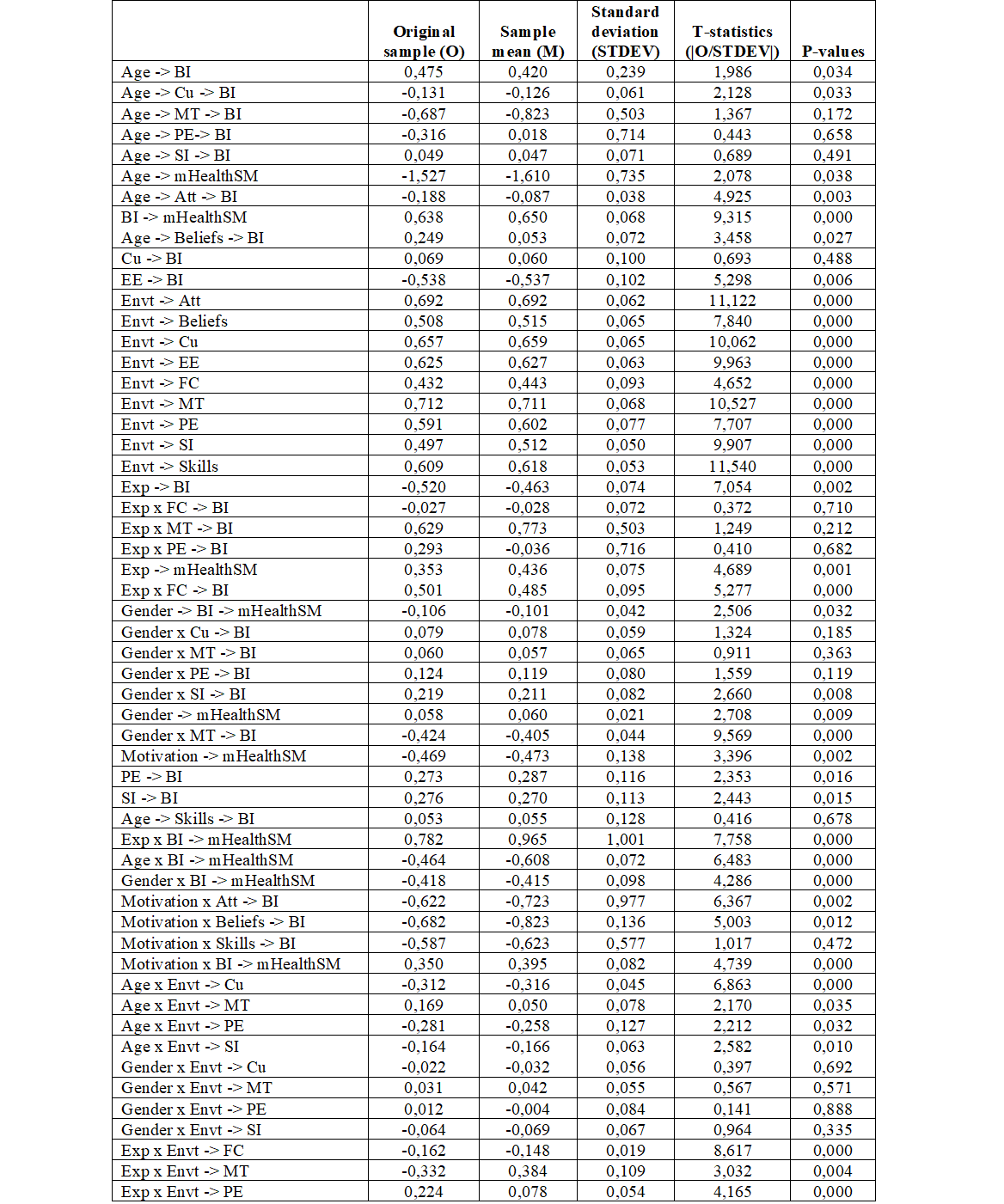

Supplement: Multimedia Appendix 1 [file formative_v7i1e49407_app1.png]
